# The effects of expectancies and patriotism on Chinese use intention of 5G network

**DOI:** 10.3389/fpsyg.2022.946000

**Published:** 2022-08-09

**Authors:** Zhang Ke, Hou Jiayi, Chen Long

**Affiliations:** ^1^School of Communication, Soochow University, Suzhou, China; ^2^Department of Psychology, Renmin University of China, Beijing, China

**Keywords:** 5G network, use intention, patriotism, Chinese user, UTAUT

## Abstract

China leads the world in the development and rollout of 5G network, yet less research has been done on the drivers of Chinese people’s adoption of 5G network, especially the specific role of national sentiments, such as the influence of patriotism on their attitude toward 5G network. The study obtained 804 effective online questionnaires from the respondents of various ages, genders, areas, and educational levels. The results based on the structural equation modeling (SEM) analysis showed that patriotism was an antecedent to Chinese users’ cognition of the conditions provided by the government and telecom operators and the extent that users were influenced by media and surrounding people, which in turn increased users’ expectations of the performance of 5G network and their confidence in adapting to 5G network, and resulted in an increased willingness to use 5G network. This research helps to understand the role of patriotism, which is an emotional factor, in stimulating Chinese users’ attitudes toward 5G network at the time when 5G network is putting into large-scale commercial use in China.

## Introduction

Advances in information and communication technologies are fundamental to the future smart cities (Javed et al., 2022). The current 4th Generation Mobile Communication Technology (4G) may not be able to satisfy the demands of users in terms of data rate, delay, and network capacity. The 5th Generation Mobile Communication Technology (5G) is becoming the focus of both industry and academia worldwide, as they are predicted to be able to deliver 1,000 times the capacity of current mobile networks, along with 10–100 times higher data rates compared to 4G wireless networks (Le et al., 2015). According to Rodriguez (2015) and Liyanage (2018), mobile stakeholders are already preparing to meet the challenging 5G requirements, such as 10–100 times more connected devices, a peak rate of up to 10GBits bits per second, 100 times more energy and cost efficiency, and 10–30 times lower latency. The benefits of deploying 5G networks are not limited to pure individual needs for lifestyle changes, but also extend to national economic, political, and military enhancement. There have been programs for developing and roll out in 5G launched worldwide, such as in Europe (e.g., 5G Public-Private Partnership Project), North America (e.g., SaT5G project), and Asia (e.g., 5G Forum in South Korea and 2020 and Beyond *Ad Hoc* in Japan). Many countries have regarded the mobile communication industry as a strategic battlefield for establishing national competitive advantage. It is no exaggeration to say that whoever dominates 5G stands on the commanding heights of the future economy.

The Chinese government regards 5G as key to China’s economic development and the development of its technology industry. The 13th 5-Year Plan described 5G as a strategic emerging industry and a new economic growth point (Eastday, 2016). The Chinese government is already determined to achieve breakthroughs in 5G mobile communication technology. During the 14th 5-Year Plan period, China is expected to complete the construction of a fully fledged 5G network, and various scenarios for 5G vertical application will be further expanded (Xinhua Net, 2021a). On June 6 of 2019, the Ministry of Industry and Information Technology of China officially issued 5G commercial licenses to China Unicom, China Telecom, China Mobile, and China Radio and Television, the four largest mobile stakeholders in the country (China Daily, 2019). By August 2021, more than 100 operators around the world have launched 5G network services, and the number of 5G users in China has exceeded 400 million (Xinhua Net, 2021b). By 2025, China is expected to become the world’s largest 5G market, accounting for one-third of global connections (Techweb. com. cn., 2018). 5G is predicted to drive China’s total output to 6.3 trillion yuan, economic value to 2.9 trillion yuan, and employment opportunities to 8 million by 2030 (Cac. gov. cn., 2017).

The impact of patriotism on user’s behavior is an important issue in user’s behavior research (e.g., Renko et al., 2012; Nik-Mat et al., 2015). The effects of national pride and national brand-consciousness of Chinese users have been explored in several studies (e.g., Wang et al., 2012; Du and Wang, 2018; Zhang et al., 2018). The commitment and determination to achieve 5G leadership from both the Chinese government and Chinese enterprises is complemented by the patriotism of the Chinese people, who regard the 5G enterprises represented by Huawei as a symbol of national pride (ChinaIT, 2018). Chinese companies such as Huawei have continued to conduct independent technology research and development and become increasingly powerful. The label of their “national enterprise” has become more and more distinct, and it has also demonstrated the national confidence that the Chinese people need (UZBCN, 2019).

This study is based on three motivations: the need of the government to arouse the enthusiasm of the public, the need of operators to stimulate the purchasing sentiment of consumers, and the need to clarify the relationship between several variables in the unified theory of acceptance and use of technology (UTAUT) model. To be specific, many countries regard 5G as the key to building competitive advantages and standing at the commanding heights of the future economy. All levels of government must face the problems of how to grab 5G development opportunities through rapidly mobilizing the enthusiasm and self-confidence of the people of the country. Meanwhile, the competition in the 5G market will be far more intense than that in previous telecommunications markets. For the purpose of surviving and reaping rewards in this increasingly fierce market environment, operators must analyze consumers’ attitudes toward the 5G network environment from the perspective of their sentiments. In addition, the effect of the four predictive UTAUT factors on behavioral intention has usually been tested separately, and very few pieces of research have explored their inner correlations.

With these motivations in mind, we aim to test the factors that influence Chinese peoples’ intention in adopting and purchasing 5G network based on integrating patriotism into the UTAUT model. This paper contributes to the following:

1. Constructing a theoretical model that explains the drivers of Chinese people’s adoption of 5G network;
2. Providing useful insights into the transformation of patriotism and national sentiments into user’s behavior intention;
3. Distinguishing between predicted factors and affected factors of the UTAUT model in terms of the Chinese people’s attitude toward the 5G network;
4. Helping governments and relevant departments to grasp user needs in the future of 5G evolution process.

## Theoretical framework and hypotheses

The existing studies on the use of communication technology mainly focused on the convergence and obedience of people to norms of social public behavior (e.g., Lu et al., 2008; Kuo and Yen, 2009) and perceptions of risk in the Internet age (e.g., Yang et al., 2012; Yan and Yang, 2015; Xiang, 2017). Research on 5G network has primarily focused on improving on 5G technology, ranging from the challenges in creating the technology itself (Jaber et al., 2016; Ge et al., 2017) and impacts of using 5G technology in various industries (Betzalel et al., 2018; Rao and Prasad, 2018) to technical security and privacy protection (Fang et al., 2018). Related research fields include U-learning (Park, 2014), smart city implementation (Rao and Prasad, 2018), persuasive business models (Lindgren, 2016), big data application (Hossain et al., 2016), and health (Di Ciaula, 2018).

### Unified theory of acceptance and use of technology and technology acceptance

The unified theory of acceptance and use of technology, proposed by Venkatesh et al. (2003) as a consolidated update on prior models of explaining new technology acceptance, is one of the most popular models when exploring users’ behavioral intention in accepting information and technology. Venkatesh’s UTAUT explained 69% of use intention in technology, which is higher than all previous individual models (Venkatesh et al., 2003). In terms of the predictors of behavioral intention in UTAUT, performance expectancies are the extent to which a person believes that using technology will help him or her to achieve benefits; effort expectancies are the perception of the convenience of using a technology; social influence is defined as the degree of an individual’s belief toward the approval of using a technology from important others; and facilitating conditions refer to an individual’s perception of the resources and enablers available to perform an action (Venkatesh et al., 2003, 2012). All four factors have been verified in many studies (e.g., Tarhini et al., 2016; Bendary and Al-Sahouly, 2018) as significantly affecting user adoption behavior.

The unified theory of acceptance and use of technology has been applied and tested in a variety of studies in the field of communication and information technology, including E-government systems and services (Alawadhi and Morris, 2008), online banking (Foon and Fah, 2011), blogging technology (Pardamean and Susanto, 2012), mobile libraries (Wang, 2017), social media (Salim, 2012), mobile electronic medical records (Kim et al., 2016), mobile commerce (Bendary and Al-Sahouly, 2018), tax payment systems (Ajunwa, 2016), clinical informatics research (Abayomi et al., 2016), and information and communication technology (Alwahaishi and Snásel, 2013). With the aim of understanding the Chinese people’s usage intentions toward the incoming 5G mobile network, this research tests the power of the four driving forces of UTAUT in predicting Chinese users’ use intention toward 5G. Therefore, the first hypothesis is proposed as follows:

H1: Chinese people’s perceived effort expectancy of using 5G (H1a), performance expectancy of 5G (H1b), and social influence (H1c) and facilitating conditions for 5G use (H1d) enhance their intention to use 5G.

### Patriotism and consumption

Patriotism represents an individual’s strong feeling of loyalty and attachment to his country without showing hostility toward other countries (Kosterman and Feshbach, 1989; Balabanis et al., 2001). A patriot is usually characterized by a willingness to make sacrifices for his country and to put his own interests behind national interest (Feshbach, 1990). Patriots typically support domestic producers out of a sense of duty and loyalty to their nation, because they perceive that this behavior can greatly influence their country (Han, 1988; Rybina et al., 2010). Past studies have found that users’ patriotism causes them to more positively evaluate or favor domestic products (Han, 1988; Renko et al., 2012; Nik-Mat et al., 2015). Chinese users’ national-brand consciousness is largely based on their national sentiments and patriotic enthusiasm (Han and Liu, 1995) and can be mapped to their national feelings, that is, their love, care, pride, and anxiety for their country in the field of consumption (Zhuang et al., 2006).

Patriotism is essentially a loyalty to the country, with an intense desire for the prosperity of the country. Subconsciously, Chinese people often associate patriotism with the rise of national brands and a strong national economy (Wang et al., 2012). The Chinese people today cherish the country’s 5G leadership position and see it as an achievement built on the tireless efforts of several generations. Their perception of the current situation is that, as the most powerful competitor in the 5G market, Huawei is frequently treated unfairly in the international arena (Global Times, 2018). The persistence and courage of Huawei in the face of unfair treatment (Iyiou, 2018) has also inspired the Chinese people’s national spirit and sense of solidarity. Chinese people from all walks of life have expressed their support for Huawei (Pan, 2019), and patriotism is rising. Huawei is the enterprise with the greatest possibility of providing China with 5G services in the near future. The Chinese people’s patriotism, inspired by the courage and effort of Huawei, has aroused their support for Huawei’s products, including Huawei mobile phones and its related 5G network (Savitz, 2019). Hence, we propose the second hypothesis.

H2: Chinese people’s patriotism enhances their intention to use 5G.

### Patriotism, facilitating conditions, and 5G expectancy

Facilitating conditions is the users’ perception of the extent to which an organization or related infrastructure supports the usage of a technology (Venkatesh et al., 2003), which is defined in this study as the layout and support for 5G networks and related facilities by organizations and institutions such as government, 5G R&D companies, and telecom operators. The more convenient these facilitating conditions are, the more confidence users have in 5G performance and the stronger their willingness to use 5G is. In China, cheap tariffs and extensive coverage have allowed the entire country, including cities and rural areas, to enter the era of mobile Internet. The Chinese government has played a crucial role in promoting the development of 4G and mobile internet services in China, with striking results. Now, the government’s attitude toward 5G development is very clear—that is, actively supporting the acceleration of 5G deployment and all-optical network construction (Gov.cn., 2018).

As the Chinese people’s patriotism rises along with the gradual increase in national strength and economic prosperity, the people’s trust in the ability in laying out infrastructure of the government and the productive ability of enterprises is increasing. According to the 2019 Edelman Trust Barometer, a report released by Edelman, the world’s largest public relations enterprise, China leads the rest of the world in terms of the Chinese people’s trust in their government, enterprises, media, business, and non-governmental organizations (Edelman, 2019). It is reasonably thought that the Chinese people trust the ability and efficiency of the government and the three telecom operators to deploy 5G nationwide.

Tu et al. (2019) demonstrated that facilitating conditions positively affected users’ effort expectancy. China’s fiberoptic broadband has the widest coverage in the world, and China’s urban areas generally have 100-megabit access capabilities (Chinanews, 2017). Chinese people believe that the country’s leaders have helped them to achieve upward class mobility and improve their quality of life (Edelman, 2019). The strong social management capabilities of the country and the capacities in laying out wireless communication technology of Chinese telecom operators will enhance the Chinese people’s expectation of stable and widespread 5G performance and will also enhance the Chinese people’s confidence in the smooth use of 5G.

Patriotism may lead the Chinese people to place more trust in the capacity and efficiency of the government and the involved enterprises in the planning and construction of 5G, may in turn increase the Chinese people’s recognition of the day-to-day benefits that 5G brings, and may improve their confidence in integrating 5G into their lives. Hence, we propose the following hypotheses:

H3: Chinese patriotism enhances their perceived facilitating conditions for 5G.

H4: Chinese perceived facilitating conditions for 5G enhance their perceived effort expectancy (H4a) and performance expectancy (H4b) of 5G.

H5: Facilitating condition mediates the relationship between Chinese patriotism and perceived effort expectancy (H5a)/performance expectancy (H5b) of 5G.

### Patriotism, social influence, and 5G expectancy

Numerous media reports about 5G have emerged since 2017. For example, reports have claimed that 5G’s high-speed and low-latency technology innovation will be interconnecting everything (Song, 2019); that the 5G network will bring a whole suite of dramatic improvements to the user’s mobile data experience (Woyke, 2018); that the development of 5G will determine the fate of many companies (OFweek, 2018); that China’s 5G is ahead of the rest of the world (Chinanews, 2017); that 5G will bring great changes to the lives of ordinary people through the widespread application of cloud technology (Oushinet, 2019); and that 5G will bring new conveniences to all aspects of life, such as medical care, education, transportation, and home (Eastday, 2019). The introduction and publicity of 5G in the media gave rise to great public expectations for the coming of 5G and also built up confidence in the planning of 5G in China.

National pride is one of the reasons why China attaches so much importance to 5G technology (Woyke, 2018). Whenever there are reports of Chinese 5G technology being suppressed in the international market or of unfair treatment of Chinese 5G companies by foreign governments, they give rise to a spirit of solidarity among the Chinese people, inspired by patriotism, prompting them to support China’s 5G network. Reports describing how the Chinese people have consistently developed an attitude of assured support to domestic 5G network can often be seen in the media (e.g., Zhao, 2019; Si, 2021). About 84% Chinese people have also said that they want to buy Huawei 5G mobile phones and support domestic production (Savitz, 2019).

Social influence refers to the user’s perception that relatives, friends, colleagues, and acquaintances feel that he or she should use a certain technology and that the media advocates the use of certain products (Min et al., 2008). This study believes that if the media and the people around the user say that 5G network is good, promoting the use of 5G, the user too will feel that 5G is useful, that it will meet his or her own performance expectations, and that he or she can enter the 5G world as smoothly as everyone else.

News about 5G or related enterprises is often seen on mass media and social media, and for the Chinese, 5G is a thing that is certain to enter everyone’s life in the near future. Across society, from government to business to media to individuals, everyone is discussing 5G (Zhu, 2018). Word of mouth has contributed to ensuring that the average Chinese person understands and is looking forward to 5G.

Patriotism may lead the Chinese people to be more influenced by the media’s promotion of China’s leading technology as well as by praise by the people around them for national enterprises such as Huawei. This in turn may increase the Chinese people’s recognition of the day-to-day benefits that 5G brings and may improve their confidence in integrating 5G into their lives. Hence, we propose our final hypotheses as follows.

H6: Patriotism enhances the extent to which social influence affects Chinese users.

H7: Social influence enhances Chinese perceived effort expectancy (H7a) and performance expectancy (H7b) of 5G.

H8: Social influence mediates the relationship between Chinese patriotism and perceived effort expectancy (H8a)/performance expectancy (H8b) of 5G.

**FIGURE 1:**
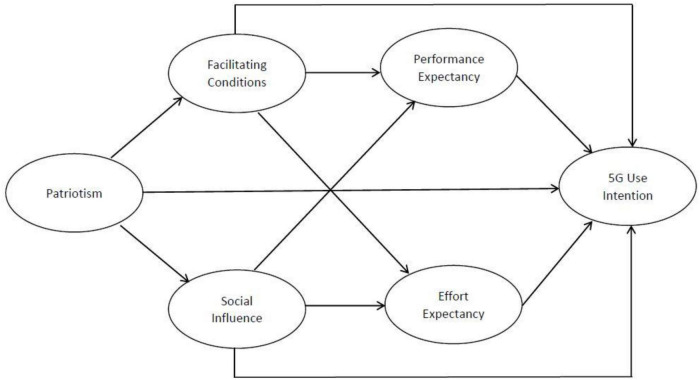
Conceptual model of Chinese 5G use intention.

The conceptual framework of this study is shown in Figure 1, based on the proposed hypotheses.

## Materials and methods

This research utilizes online survey to collect the self-report data of Chinese users, for the purpose of understanding their perception of expectancy brought by 5G effectiveness and its related external environment as well as their willingness in purchase and use 5G network.

### Measures

Patriotism (α = 0.881) was adapted from Kosterman and Feshbach (1989). Purchase and use intention (α = 0.849) of 5G network and its four predictors in UTAUT, namely, effort expectancy (α = 0.792), performance expectancy (α = 0.809), social influence (α = 0.815), and facilitating conditions (α = 0.746) were adapted from (Venkatesh et al. (2003)). To ensure structural equivalence, the measurement items were translated into Chinese and then back-translated into English. Details of the measures are shown in Table 1.

**TABLE 1:** Measurement items and standardized factor loading.

*Patriotism*	SFL
1. I am emotionally attached to China and emotionally affected by its actions.	0.653
2. I feel strong ties with China.	0.638
3. When a foreigner praises China, it feels like a personal compliment to me.	0.663
4. The fact that I am Chinese is an important part of my identity.	0.657
5. I am proud to be a Chinese.	0.703
6. It is important for me to serve China.	0.660
7. I am willing to live in China forever.	0.661
8. I think China and people are the best.	0.671
9. The primary responsibility of every young person is to respect the history and traditions of China.	0.688
***Facilitating conditions***	
1. Telecom operators will serve people to access 5G.	0.690
2. The government and relevant departments will help people understand and use 5G.	0.678
3. The government and telecom operators will soon deploy facilities that support 5G nationwide.	0.616
4. Enterprises will soon develop and popularize products and equipment for 5G.	0.626
***Social influence***	
1. People around me agree and support the use of 5G.	0.662
2. Expressing the willingness to use 5G will allow me to get more recognition from the people around me.	0.656
3. Using 5G will allow me to be better accepted by the surrounding groups.	0.637
4. Promotions and advertisements related to 5G will prompt me to use it.	0.660
5. Stories of public figures related to 5G will prompt me to use 5G.	0.622
6. The government’s policies of encouraging the use of 5G will prompt me to use 5G.	0.651
***Performance expectancy***	
1. The use of 5G improves the efficiency of my work and life.	0.706
2. Using 5G improves the satisfaction of my mobile phone usage.	0.677
3. Using 5G makes my life easier.	0.669
4. Using 5G makes it easy for me to access the information I need.	0.657
5. Using 5G adds to the fun of my life.	0.677
***Effort expectancy***	
1. It is easy to learn how to use 5G services.	0.653
2. I don’t need to work hard to use 5G services.	0.603
3. 5G mobile phone system is very easy for me to operate.	0.640
4. I can easily adapt to 5G networks.	0.688
5. I can easily adapt to the world of all things connected brought by 5G.	0.696
***Use intention***	
1. I am looking forward to the massive use of 5G networks.	0.691
2. I am looking forward to the emergence of 5G related products on the market.	0.673
3. I would like to learn how to use 5G network.	0.717
4. I will use 5G services.	0.705
5. I will try 5G related products.	0.700
6. I would recommend other people to use 5G.	0.676

N = 840.

### Data collection

The study was conducted on WJX, China’s largest non-proprietary online survey platform, during July and August in 2020. WJX has an e-panel comprised of more than 2.6 million members. To avoid repeats, respondents with the same computer/mobile phone can access the survey one time only. A total of 804 respondents, including 421 females and 383 males, completed the questionnaire. Almost half of the respondents (*n* = 390) were young people between 18 and 32 years old, and one quarter of them (*n* = 204) were middle-aged people between 33 and 47 years. About four-fifth of respondents (*n* = 638) were well educated, with a college’s degree or above. More than one-third of respondents (*n* = 300) and one-quarter of them (*n* = 201) live in the first-tier and second-tier cities, respectively. Almost one-third of the respondents (*n* = 255) have experienced 5G network.

## Results

### Hypothesized path model test

Structural equation modeling (SEM) approach by Mplus was used to test the proposed model (Singh, 2009). The model generated in this study reached good data-model fits suggested by Bagozzi and Yi (2012). We began by examining the effects of four predictors of UTAUT on Chinese purchase and use intention of 5G network. Results show that both effort expectancy (β = 0.125, *p* = 0.006) and performance expectancy (β = 0.561, *p* = 0.010) have significant effect on Chinese users’ purchase and use intention of 5G network, supporting H1a and H1b. But contrary to H1c and H1d, neither social influence (β = –0.008, *p* = 0.964) nor facilitate conditions (β = 0.117, *p* = 0.450) could directly affect 5G usage intention.

Following, we tested the effects of patriotism on social influence and facilitation conditions. In support of H3 and H6, Chinese patriotism exerts significant effects on their perceived facilitating condition for 5G (β = 0.927, *p* = 0.000) and the extent to which social influence affects them (β = 0.870, *p* = 0.000). Meanwhile, patriotism could not directly affect Chinese usage intention of 5G (β = 0.197, *p* = 0.103), and H2 cannot be supported.

**FIGURE 2:**
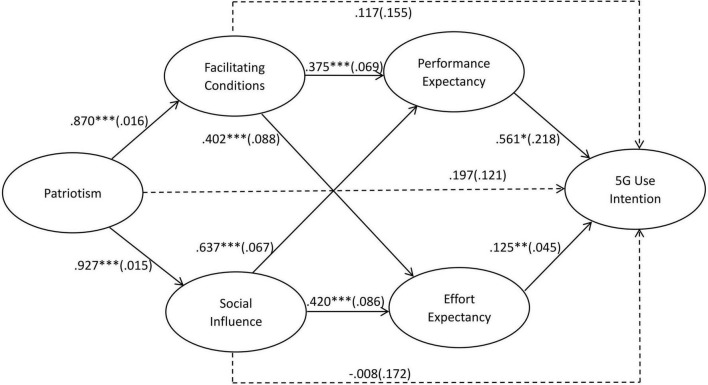
Statistical model of Chinese 5G use intention. *N* = 804. *p < 0.05, **p < 0.01, ***p < 0.001. Model fit, Chi-square value X^2^ (549) = 1848.163, RMSEA = 0.054, CFI = 0.903, SRMR = 0.036.

Then, we examined the effects of social influence and facilitating conditions on effort expectancy and performance expectancy separately. In support of H4, Chinese perceived facilitating conditions exert positive effects on both effort expectancy (β = 0.402, *p* = 0.000) and performance expectancy (β = 0.375, *p* = 0.000). Meanwhile, in support of H7, the extent to which social influence affects Chinese exerts positive effects on both effort expectancy (β = 0.420, *p* = 0.000) and performance expectancy (β = 0.637, *p* = 0.000). The results of the standardized model are shown in Figure 2.

### Mediation effects test

Mplus was utilized to implement the mediation effects test. Then, 5,000 bootstrap samples recommended by Cheung and Lau (2008) were used. The results show that the 95% BC confidence interval of the mediation effect from patriotism to effort expectancy *via* facilitating conditions did not include zero (lower 2.5% limit = 0.116; upper 2.5% limit = 0.589) and the 95% BC confidence interval of the mediation effect from patriotism to performance Expectancy *via* Facilitating Conditions did not include zero (lower 2.5% limit = 0.104; upper 2.5% limit = 0.551), in support of H5, that facilitating conditions mediates the relationship between patriotism and effort expectancy/performance expectancy.

Likewise, the 95% BC confidence interval of the mediation effect from patriotism to effort expectancy *via* social influence did not include zero (lower 2.5% limit = 0.125; upper 2.5% limit = 0.567) and the 95% BC confidence interval of the mediation effect from patriotism to effort expectancy *via* social influence did not include zero (lower 2.5% limit = 0.327; upper 2.5% limit = 0.745), in support of H8, that social influence mediates the relationship between patriotism and effort expectancy/performance expectancy. Table 2 shows the output file with details of the estimated mediation effects and BC bootstrap confidence intervals.

**TABLE 2:** Confidence intervals of standardized mediation effects.

Confidence intervals of standardized total indirect effects, specific indirect effects, and direct effects							
	*L.*5%	*L* 2.5%	*L 5%*	Estimate	*U 5%*	*U 2.5%*	*U.5%*
**Effects from PA to EF**							
Sum of indirect	0.642	0.665	0.677	0.738	0.800	0.812	0.835
Specific indirect							
EF							
SI							
PA	0.050	0.125	0.164	0.365	0.567	0.606	0.681
EF							
SI							
PA	0.035	0.116	0.157	0.373	0.589	0.630	0.711
**Effects from PA to EF**							
Sum of indirect	0.840	0.855	0.862	0.902	0.941	0.949	0.964
Specific indirect							
EF							
SI							
PA	0.256	0.327	0.364	0.554	0.745	0.782	0.853
EF							
SI							
PA	0.028	0.104	0.143	0.347	0.551	0.591	0.667

Bootstrap, 1,000; L, lower; U, upper; PA, patriotism; EX, effort expectancy; PE, performance expectancy.

### Multi-group analysis

Finally, this research tested whether there were significant differences in the hypothesized path model in respondents with/without experience of using 5G network, to help understand whether the proposed model could be used to understand the true intention of users when 5G is put into large-scale commercial use in China in the future.

To compare these two groups, a multi-group analysis by Mplus was performed. In this analysis, each causal path was constrained equally across both groups at a time, and a model with each constrained path was compared with the completely unconstrained model. If the AIC and BIC values of the constrained model are significantly worse than that of the unconstrained model, the constrained path coefficients are identified as significantly different between the two groups (Kline, 1998). For AIC and BIC information criteria, lower values indicate improved model fitting (Burnham and Anderson, 2004; Vrieze, 2012).

The results show that the fitting information of the SEM model with constrained regression coefficients [AIC = 81554.743, BIC = 82319.147] is lower than that of the model with unconstrained regression coefficients [AIC = 83127.118, BIC = 83671.112]. It means that there is significant difference between people with/without 5G usage experience in terms of extent of the proposed model in explaining their 5G usage intention. It indicates that proposed model based on the perspective of the combination of UTAUT and patriotism is more suitable for the people who have real usage experience of 5G network.

### Summary of results

From the above analysis, we can draw the conclusion that the patriotism of Chinese people improves their perception of the facilitating conditions and social influence of 5G network. In addition, both social influence and facilitating conditions are drivers of Chinese people’s perceived performance expectancy and effort expectancy of using 5G network, and the latter two variables are predictors of their purchase and use intention toward 5G network.

Further, social influence and facilitating conditions separately mediate the relationships between patriotism and performance expectancy/effort expectancy. This indicates that patriotism first enhances Chinese people’s perception of the facilitating conditions and the extent to which social influence affects them, in turn strengthening their expectations of the performance of 5G and of their ability to adapt to 5G network.

## Discussion

This research shows that, in China, patriotism first affects people’s perception of government and enterprise capabilities and the extent to which they are influenced by the media and people around them. This in turn affects expectations of 5G’s function and expectations for adapting to 5G and ultimately affects 5G use intentions.

### Mechanism of patriotism as rootage in stimulating Chinese use intention of 5G

The complete path from patriotism to purchase and use intention of 5G network, as verified in this research, provides a full understanding of how Chinese patriotism exerts an influence on the four drivers of UTAUT, which in turn leads to usage intention of 5G network. The mechanism can be explained as follows. Feelings of patriotism improve the trust that Chinese people have in the facilitating conditions provided by the government, technology enterprises, and telecom operators and enhance the extent to which people are influenced by media and surrounding people in terms of promoting 5G network and its related products. This in turn strengthens people’s confidence in adapting to the future 5G world and their trust in the performance of 5G network. Finally, people’s willingness to use 5G network is strongly based on their high expectancy.

Patriotism is a strong feeling that has long been inherent to the Chinese nation. Specifically, for Chinese citizens who have suffered and persevered during the past 100 years, patriotism is a potent agent with the power to influence society and current events. The Chinese people’s strong expectation that 5G will deliver convenience and other benefits to their life and their confidence in their ability to integrate into a world where all things are connected by 5G are both inseparable from the patriotism and national pride inspired by the country’s strength and economic prosperity.

Patriotism, which is complemented by national pride and a spirit of working for national advancement, makes Chinese people believe that the government and relevant institutions will successfully deploy 5G facilities nationwide. The strong social management capabilities of the country and the Chinese telecom operators enhance the Chinese people’s recognition of the stability and wide coverage of 5G as well as their confidence in its smooth usability.

Patriotism also renders them more susceptible to promotion of 5G network in the media and by word-of-mouth. The 5G enterprises represented by Huawei often emphasize the struggle for national interest in its public relations strategies which greatly incites the patriotism of the people. For example, since the first half of 2019, the US-sponsored trade sanctions against Huawei have become one of the hottest topics on the Internet in the country of the whole network, one that has quickly stimulated the nationalist sentiments of the Chinese people and contributed to the improvement of Huawei’s local reputation (UZBCN, 2019).

### Theoretical contributions

This paper presents new insights into the extent to which patriotism can be transformed into user intentions. Specifically, this paper uses empirical methods to verify the antecedent variables of 5G use intentions in China’s domestic environment, including patriotism and UTAUT. It embeds Chinese patriotism into the evaluation and perception of the functional utility and external environment of 5G in China, establishing a UTAUT-based use intention evaluation model, helping explore the factors affecting Chinese users’ use intention of the coming 5G networks, and providing a new perspective for research into 5G services.

This study found that social influence and facilitating conditions can both be the drivers of the two aspects of expectancy. It indicates that, on the one hand, Chinese people’s expectation and estimation of an upcoming large-scale universal technology are boosted by their cognition of the capacity and effectiveness of the government and national enterprises, and on the other hand, the Chinese are easily influenced by the opinions of the people around them and also trust the media when considering the adoption of a technology that is closely related to all aspects of life. This is an innovative restructuring of the UTAUT, providing new evidence for future research based on this concept.

### Practical implications

This study collects empirical data on user’s perceptions of 5G in terms of government layout, corporate R&D, media promotion, operator building, and 5G usage intentions for understanding and predicting the future use of 5G networks in a large scale of people. The study provides decision-making references and data support for 5G use in various fields.

In terms of facilitating conditions and social influence, operators can organize various activities to promote 5G technologies and related products in society, to introduce the functions of 5G networks, and to enable the public to fully understand the improvements in quality of life that 5G technologies may bring. Governments and operators can encourage people to recognize and adopt 5G network through word-of-mouth communication on media and in real life. At the same time, 5G R&D companies can strengthen cooperation with governments and telecom operators to expand the uses of 5G network, such as developing 5G-related technologies for education, medical care, old-age care, transportation, and other fields. In addition, industry R&D into 5G network can strengthen connections with 5G-related products and organizations, develop uses and products related to 5G in all aspects of life, and promote the accessibility of 5G technologies in various fields.

## Conclusion and suggestions for future research

The localization research carried out in this paper provides useful insights into the transformation of patriotism and national sentiment into consumer’s behavior intentions. Users’ willingness to adopt 5G technology and related products involves complex psychological behavior, which needs to be systematically researched in multiple dimensions. The future research could test more complex variables that may have effects in stimulating consumers in adopting and using network technology. In addition, with the large-scale commercialization of 5G networks in more and more countries, studies examining the relationship between patriotism and consumer’s purchase intentions or behaviors should consider the effects of brand characteristics (such as the quality and cost performance of different brands of mobile phones supporting 5G), to obtain information on consumer attitudes that are more relevant to national markets.

## Data availability statement

The data that support the findings of this study are available from the ZK, upon reasonable request.

## Author contributions

All authors listed have made a substantial, direct, and intellectual contribution to the work, and approved it for publication.
